# Linear array of conserved sequence motifs to discriminate protein subfamilies: study on pyridine nucleotide-disulfide reductases

**DOI:** 10.1186/1471-2105-8-96

**Published:** 2007-03-16

**Authors:** César L Avila, Viviana A Rapisarda, Ricardo N Farías, Javier De Las Rivas, Rosana Chehín

**Affiliations:** 1 Departamento Bioquímica de la Nutrición, Instituto Superior de Investigaciones Biológicas (CONICET-UNT) and Instituto de Química Biológica Dr Bernabé Bloj, Chacabuco 461 (4000), San Miguel de Tucumán, Tucumán, Argentina; 2 Instituto de Biología Molecular y Celular del Cáncer (IBMCC-CIC, CSIC/USAL) Campus Miguel de Unamuno s/n. Salamanca, Spain

## Abstract

**Background:**

The pyridine nucleotide disulfide reductase (PNDR) is a large and heterogeneous protein family divided into two classes (I and II), which reflect the divergent evolution of its characteristic disulfide redox active site. However, not all the PNDR members fit into these categories and this suggests the need of further studies to achieve a more comprehensive classification of this complex family.

**Results:**

A workflow to improve the clusterization of protein families based on the array of linear conserved motifs is designed. The method is applied to the PNDR large family finding two main groups, which correspond to PNDR classes I and II. However, two other separate protein clusters, previously classified as class I in most databases, are outgrouped: the peroxide reductases (NAOX, NAPE) and the type II NADH dehydrogenases (NDH-2). In this way, two novel PNDR classes III and IV for NAOX/NAPE and NDH-2 respectively are proposed. By knowledge-driven biochemical and functional data analyses done on the new class IV, a linear array of motifs putatively related to Cu(II)-reductase activity is detected in a specific subset of NDH-2.

**Conclusion:**

The results presented are a novel contribution to the classification of the complex and large PNDR protein family, supporting its reclusterization into four classes. The linear array of motifs detected within the class IV PNDR subfamily could be useful as a signature for a particular subgroup of NDH-2.

## Background

### Sequence information and protein function

One of the main aims of computational biology is to infer protein function using sequence information. Clustering of proteins in families sharing functional characteristics and derived from a common ancestor is a key purpose of sequence comparative analyses. However, algorithms used to explore sequence similarity and to retrieve homologous proteins, such as BLAST [1] or FASTA [2], are not sensitive enough to find out evolutionary divergent members of large protein families, while PSI-BLAST [3] increases the sensitivity on detriment of specificity. In this scenario, an efficient strategy to detect more divergent sequences within protein families is the use of sequence motifs. These are highly conserved regions across a subset of proteins sharing the same function. In general, they play an important role in protein functions and folds [4]. Furthermore, several motifs may be arranged into fingerprints which improve the detection of remote homologous proteins reducing the "noise" that accompanies the local alignment algorithms.

### Databases of motifs and domains

The most widely used biological databases that explore and classify proteins according to their composition in patterns, motifs or domains are: PROSITE [5]; Pfam [6]; BLOCKS [7]; PRINTS [8] and InterPro [9]. InterPro is at present the database that includes the widest and most comprehensive classification of proteins families since it integrates the information about domains and motifs from most of the other databases [9]. While all of the resources share a common interest in protein family classification using sequence similarity as a key factor to achieve their purposes, the focus of each database is different. In fact, the specific methods underpinning each of them are optimal for different purposes. Some databases use all-encompassing domain/profile-based approaches and they are good to detect members of divergent superfamilies (*i.e*., Pfam). Others use motif-based approaches which correspond to functional sites, being appropriated to detect members of more specific subfamilies (*i.e*., PROSITE and PRINTS). Several of these databases also include structural information useful for the identification of globular protein domains. In general, the protein families proposed are quite large, having many members with rather different biochemical functions and activities. Therefore, it is important to try new ways to achieve an improved functional assignment within the protein families.

### The PNDR protein family

The pyridine nucleotide disulfide reductases (PNDR) is a large and heterogeneous protein family with a characteristic disulfide redox-active site together with the NAD(P)H and FAD binding sites [10]. In InterPro the PNDR includes around 10,000 proteins divided in two main subfamilies: class I (IPR001100) with 6,701 sequences and class II (IPR000103) with 2,809 sequences. These two large classes are further divided in other subfamilies that incorporate subsets of proteins with some specific motifs or domains. For each subset, a different InterPro accession number is given. More restrictive motifs in order to place a protein in the PNDR family are the "active site class I" and "active site class II", bearing InterPro IDs: IPR012999 (1,608 sequences) and IPR008255 (701 sequences), respectively.

Since most proteins have multiple domains and motifs and InterPro give a particular assignment for each of them, the highly conserved modules (domains or motifs) impose strong bias in the protein classifications. In the case of the PNDR superfamily, all of the proteins include one NAD^+ ^binding motif and two FAD binding motifs. These highly conserved motifs bring together a large amount of nucleotide oxidoreductases that many times have quite different functional activities. In contrast to all the InterPro PNDR class I and class II hits, only a very small subset of proteins are included in PROSITE and in PRINTS. The PROSITE's active site pattern defined as PNDR class I (PS00076) includes 124 proteins and class II (PS00573) 73 proteins. The PRINTS' signature defined as PNDR class I (PR00411) includes a true set of 102 sequences and class II (PR00469) 41 sequences.

Type II NADH dehydrogenase (NDH-2) is a member of the PNDR family that catalyzes the electron transfer from NAD(P)H to quinones without energy-transduction. A large number of organisms, ranging from archaea to eukaryotes, present NDH-2 besides the canonical rotenone-sensitive type I NADH dehydrogenase [11]. The NDH-2 enzyme could be considered a redundant protein, but it acquires other roles in certain organisms in some conditions. For example, in *Escherichia coli*, NDH-2 has Cu(II)-reductase activity [12] rendering the cells more stable in front of high or very low copper concentrations in the culture media [13]; in *Azotobacter vinelandii*, it protects the nitrogenase complex against O_2 _inhibition [14]; in *Methylococcus capsulatus*, it mediates the electron transfer to the membrane-bound methane monooxygenase [15]. This plasticity could be associated with the presence of some specific functional motifs.

### Linear array of motifs to classify the PNDR protein family

Considering that only a small proportion of the proteins clusterized as PNDR class I and PNDR class II in InterPro have a PROSITE's PNDR active site signature, a revision of this superfamily classes was performed. To achieve this, we applied a workflow to the whole PNDR family that resulted in the identification of two new outgroups: class III and class IV. The specific linear array of motifs provides a good fingerprint to describe each class. Furthermore, one of these fingerprints aided to the analysis of PNDR class IV leading to the discovery of a new linear motif that could explain the observed Cu(II)-reductase activity in a subset of the NDH-2 proteins.

## Results and Discussion

### Identification of four classes within the PNDR family

In order to achieve a comprehensive classification of the PNDR family the following workflow was designed and performed:

**1.- **All the sequences annotated as PNDR in UniProt/SwissProt database were extracted and grouped into eleven initial protein groups or clusters based on their biochemical function: AHPF, bacterial alkyl hydroperoxide reductases; DHNA, NADH dehydrogenases or alkyl hydroperoxide reductases; DLDH, lipoamide dehydrogenase; GSHR, glutathione reductase; MERA, mercuric reductase; NAOX, NADH oxidase; NAPE, NADH peroxidase; NDH-2, NADH dehyrogenase-2; TRXB, prokaryotes, archaea and lower eukaryotes thioredoxin reductases; TRXR, higher eukaryotes thioredoxin reductases; TYTR, trypanothione reductase.

**2.- **For each one of the eleven groups a multiple sequence alignment (MSA) was built and a HMM profile was derived [16]. The number of proteins included in each MSA was: 5 AHPF, 41 DLDH, 25 GSHR, 13 MERA, 36 TRXB, 8 TRXR, 5 TYTR. UniProt/SwisProt has not enough DHNA, NAOX, NAPE or NDH-2 sequences to construct a HMM profile. In order to obtain at least 5 candidates for these groups, manual retrieval of the most referenced sequences in UniProt/TrEMBL was performed.

**3.- **To enrich the MSA of the eleven groups, new sequences were extracted from UniProt/TrEMBL database using a two-way search method: **(i) **the database was scanned with the eleven HMM profiles; **(ii) **the newly found sequences were then compared with all the groups using a second round of BLAST and they were assigned to a given group according to the lowest BLAST E-value found. The number of proteins of each final cluster after filtering out the redundancy was: 43 AHPF, 21 DHNA, 97 DLDH, 71 GSHR, 60 MERA, 61 NAOX, 11 NAPE, 56 NDH-2, 99 TRXB, 69 TRXR, 20 TYTR.

**4.- **Each cluster was submitted to MEME [17] for the detection of conserved blocks. All MEME parameters were set as default except for the maximum number of motifs which was established in 10, based on the number of motifs present in the PNDRDTASE I (PR00411) and the PNDRDTASE II (PR00469) fingerprints from PRINTS database.

**5.- **The groups were then globally analyzed by comparison of all the conserved blocks using LAMA tool [18]. Sequences with most of their blocks in common were regrouped together and sent back to step 4 up to reach stable groups.

**Figure 1:**
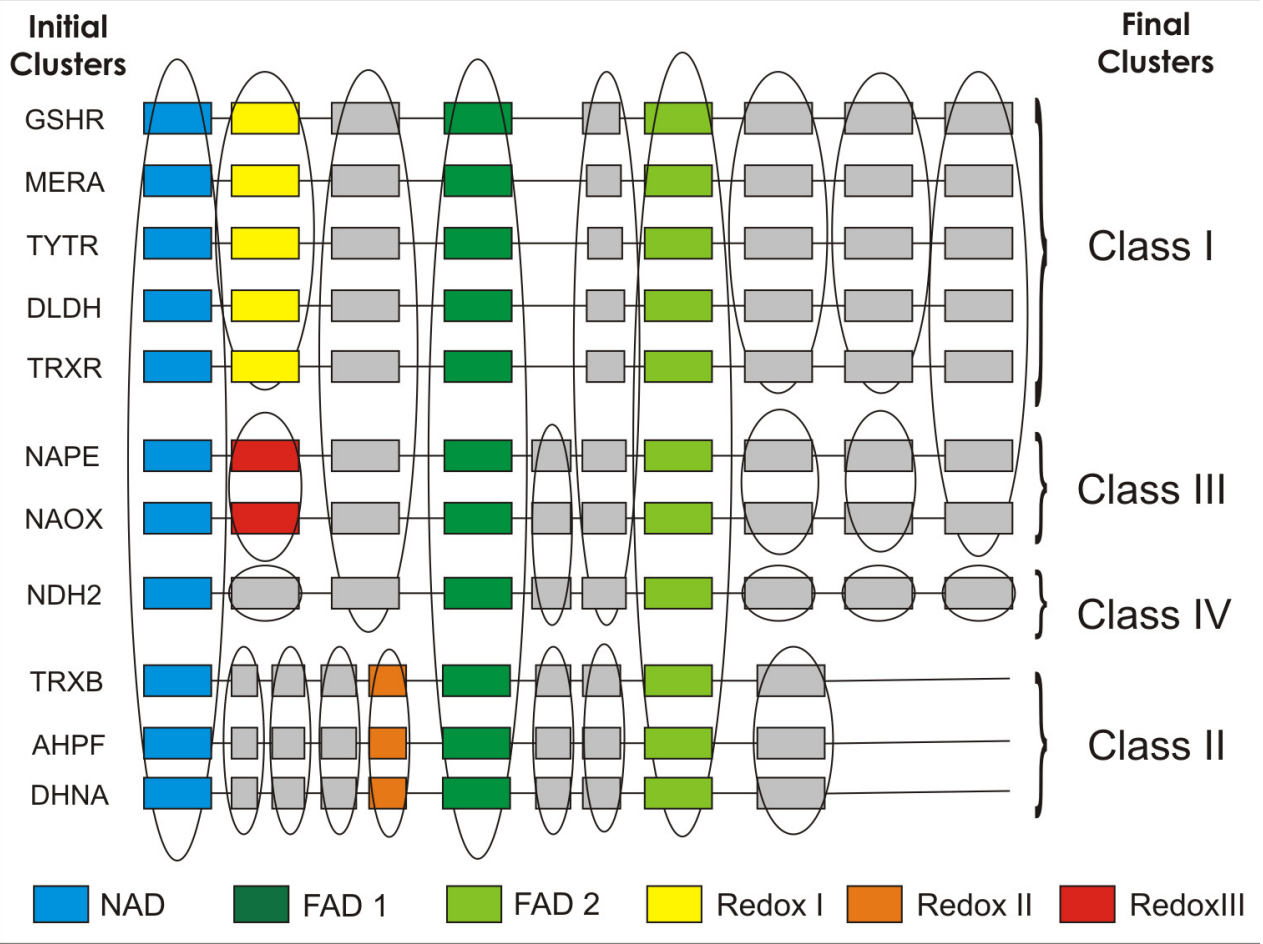
**Linear array of conserved motifs in PNDR family**. A diagram for each initial protein cluster is presented in MAST-style. Blocks with known function are depicted in color as follows: (blue) NADH-binding site; (green) FAD-binding sites; (yellow, orange and red) disulfide redox active sites for class I, II and III, respectively. The common blocks between different clusters are shown encircled, and a total of 24 different blocks are included. Names at the left indicate the 11 initial protein groups and at the right the 4 final clusters. The scheme does not represent the real length of the sequences.

The application of the above workflow resulted in 24 different conserved blocks allocated along the initial eleven protein clusters (Figure 1). All clusters share the NAD, FAD1 and FAD2 binding motifs. The workflow detected two large groups: the first one includes DLDH, GSHR, MERA, TRXR, and TYTR and the other one AHPF, DHNA, and TRXB. The fingerprints obtained for these groups were similar to the previous PRINTS annotation PR00411 and PR00469 for class I and II PNDR, respectively. This fact provides a good validation for the methodology. Additionally, two novel clusters were segregated: peroxide reductases (NAPE and NAOX) and NADH dehyrogenases-2 (NDH-2). These groups include some distinctive blocks and thus they could be classified separately as classes III and IV, respectively. The fingerprints obtained for each of the four final classes were compiled in BLOCKS format [7] and included in the Additional file 1, 2, 3 and 4.

### Phylogenetic analysis of the PNDR sequences

**Figure 2:**
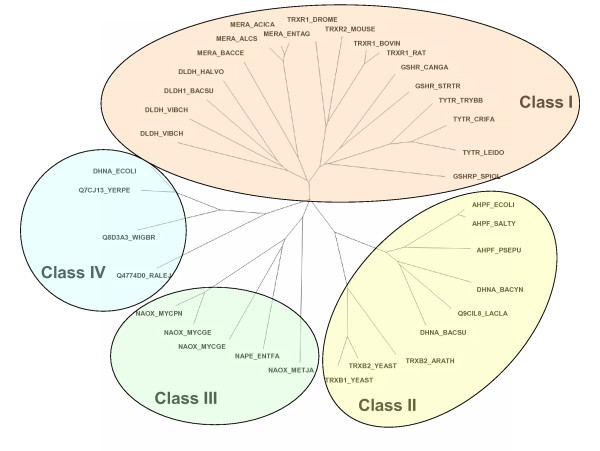
**Tree of the sequence relationship among 44 selected PNDR members**. Class I, class II, and the proposed class III and class IV segregate in 4 different central branches. The tree was obtained using neighbor-joining analysis of a subset of 44 random selected proteins sequences, using the distance matrix of the MSA built with ClustalX. The tree was visualized by TreeView software.

In order to cross-validate the proposed classification, phylogenetic sequence analysis of the PNDR protein family was performed using random selected sequences from each of the eleven protein groups. The distance matrices derived from the MSAs were analyzed using two different methods: neighbor-joining (corresponding to unrooted tree in Figure 2) and parsimony (not shown). The sequences were always segregated into four main branches, matching the classification derived from the fingerprint analysis. It is important to note that, at present, UniProt uses DHNA nomenclature to designate two types of enzymes: the alkyl hydroperoxide reductases from *B. subtilis *and *Bacillus sp*. (DHNA) and the NADH dehydrogenases-2 from *E. coli *and *H. influenzae *(NDH-2). In Figure 2 these enzymes segregate separately as expected, despite the fact that they have the same name in UniProt.

### PNDR classes III and IV

The NADH peroxidases (new PNDR class III) are structural homologues of GSHR but they do not contain a C-x(2,4)-C redox active motif [19]. Studies on NAPE from *Enterococcus faecalis *demonstrated that a modified single Cys residue (Cys-sulfenic acid, Cys42-SOH) plays the catalytic redox role. In the same way, NAOX from *E. faecalis *has mechanistic and structural similarities [20].

The type II NADH dehydrogenases (new PNDR class IV) correspond to a group of proteins widely spread in nature. Only some of them have a C-x-x-C motif, but there is little information about its biochemical function. In NDH-2 from *E. coli *the C-x-x-C motif seems to be involved in copper binding and it has been related to the Cu(II)-reductase activity of this enzyme [21].

The fingerprint based PNDR clusterization proposed above is in agreement with the classical classification criteria, which assume that these enzymes were originated by divergent evolution from an ancestral FAD/NAD(P)H oxidase and they had acquired their disulfide reductase activities independently. The segregation of the PNDR family in four classes with clear differences in their disulfide redox active sites is in support of an independent evolutionary trail.

### Specific linear motifs detected in NDH-2 PNDR class IV

**Figure 3:**
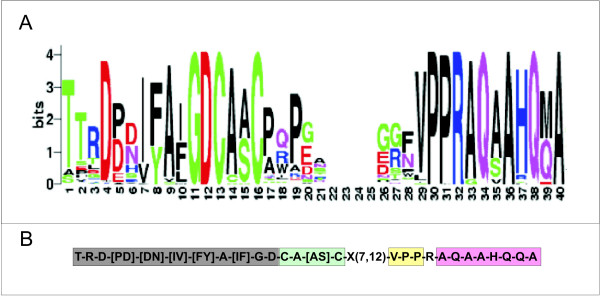
**NDH-2 conserved region around the CxxC motif**. **(A) **MSA displayed as LOGO representation of a 78 PNDR class IV subset with putative Cu(II)-reductase activity. (**B) **Derived motifs signature written in PROSITE pattern form. The shadowed blocks represent conserved regions with putative functional assignment. Grey, cyan, yellow and magenta colors correspond to: the second FAD, Cu(I), Cu(II) and quinone binding motifs, respectively.

Considering that the Cu(II)-reductase function proven in *E. coli *may be extended to NDH-2 from other organisms, a specific search to detect all the putative Cu(II)-reductases within this PNDR subfamily was performed. To achieve this, the NDH-2 subfamily was subjected to a workflow detailed as follow. First, the NDH-2s homologues were extracted from the non-redundant protein database (nrdb) using MAST [22] with the novel array of motifs developed for NDH-2 PNDR class IV (Figure 1 and additional file 4) obtaining about 500 sequences. Second, the sequences lacking the **C-x-x-C **motif from this dataset were discarded, recovering 120 sequences. Third, the redundancy was filtered out and a final group of 78 sequences was obtained. This final group of selected proteins was then analyzed by three independent motif discovery methods: MSA manual supervised inspection, MEME and PRATT automatic algorithms assessment [17,23]. The consensus regions obtained by the three methods were selected. The well-known **G-x-G-x-x-G **nucleotide (NAD^+^, FAD) binding motifs were clearly found but they were not considered. Excluding those blocks, the most conserved region found was around the **C-x-x-C **motif. The MSA of such conserved region is shown in a LOGO representation [24] in Figure 3A. The LOGO shows the residue conservation pattern for the selected region in 78 sequences, which is divided in two parts (from position 1 to 16 and from 29 to 40) and includes several putative functional motifs. Within the first part, the region between positions 1 to 12 matchs the flavin binding motif described by Eggink *et al*. [25]. The conserved C-x-x-C, located consecutive to the *Eggink's *motif, is a putative Cu(I)-binding motif [21]. The second conserved part, from position 29 to 40, includes an invariant VPP motif, which could be good candidate for a Cu(II)-binding motif considering experimental evidences from a muscle protein [26]. In addition, Cu(I) and Cu(II) putative binding motifs are separated by a loop of non-conserved residues. This kind of arrangement for copper binding has been experimentally detected in CopC, a periplasmic enzyme from *Pseudomonas syringae *[27]. Quinones also play a crucial role in NDH-2 enzymatic activity [11] and therefore, the presence of a quinone binding motif is expected. In the defined conserved region, positions 33 to 40 resemble a quinone-binding motif similar to the one proposed by Fisher *et al*. in bacterial photosynthetic reaction center [28]. In summary, the close association of the FAD, and the putative Cu(I), Cu(II) and quinone binding motifs provide an adequate arrangement for an efficient electron transfer from NADH to copper via FAD and/or quinone. The four described motifs are assembled into a signature (Figure 3B) that describes a subset of proteins from class IV PNDR related to copper metabolism.

Using this signature as a search motif, many other NDH-2s from bacteria that resist high copper concentrations were retrieved: *Pseudomonas putida *(57% ID against *E. coli *NDH-2), *Pseudomonas syringae *(54% ID), *Ralstonia sp*. (48% ID), *Salmonella enterica serovar Typhimurium *(97% ID),*Erwinia amylovora *(80% ID), *Cupriavidus necator *(50% ID) [29-33]. Since the pair-wise identity (ID) in the full sequences alignment ranges from 99 % to 22 %, the conserved region found is considered statistically significant and therefore it can be related to a specific function within the NDH-2 subfamily. High conservation of this region reflects a relatively high selection pressure through the evolution. Further experimental studies will be needed in order to test the proposed functional assignment predicted for the NDH-2 proteins.

## Conclusion

The wide use of protein sequence analysis together with the exponential growth of protein databases justify the demand of new approaches to protein comparison and functional classification. The presented strategy proves to be efficient to perform protein clusterization based on the assignment of fingerprints (*i.e*. linear array of conserved motifs and domains), since it allows to recognize divergent members within large protein families. The proposed method is applied to the complex and large PNDR protein family improving its classification and detecting two non-described outgroups. Moreover, a specific array of motifs detected in a subset of proteins within class IV PNDR subfamily is proposed as a new signature for NDH-2 proteins that may have copper binding-redox activity.

## Methods

In order to automate data processing, standard bioinformatics tools in addition to a few home-built PERL scripts were used.

### Construction and matching up of PNDR family fingerprints

Initial protein clusters were built using keyword search in UniProt/SwissProt database. MSA were generated using ClustalX [34] with default parameterization. HMM profiles [16] were created with HMMER package [35]. Initial clusters were further enriched implementing a reciprocal best-match approach (*i.e*. extracting sequences from UniProt/TrEMBL based on a HMM profile search and performing the reverse BLAST [1] search of each sequence against all clusters). Conserved blocks among each cluster were detected by MEME [17] setting the maximum number of blocks to 10 and all other parameters as default. Block to block comparison was performed using LAMA [18]. Clusters having more than 8 blocks in common were fused together.

### Phylogenetic studies

Unrooted tree for the PNDR family was generated with four randomly selected sequences from each of the initial clusters, *i.e*. AHPF, DHNA, DLDH, GSHR, MERA, NAOX, NAPE, NDH-2, TRXB, TRXR, TYTR. The neighbor-joining analysis was performed from a MSA with default parameterization and with 1000 bootstrap trials using ClustalX [34]. The results were visualized with TreeView [36]. The maximum parsimony method was carried out using PHYLIP package [37].

### Designing functional protein signature related to copper metabolism in NDH-2

NCBI's non-redundant database (nrdb) was scanned for NDH-2s using MAST [22] fed with the PNDR class IV fingerprint with a 10^-25 ^E-value cut-off. Only sequences bearing a C-x-x-C motif were kept and redundancy was further removed based on a 90 % sequence identity criteria. Motifs were extracted using PRATT [23] and MEME [17] automatic algorithms, and further reinforced by manual inspection of the MSA. Pattern based searches were performed using ScanProsite standalone tool [38]. The LOGO representation of sequence conservation was created by WebLogo [39].

## List of abbreviations used

MSA, Multiple sequence alignment; HMM Hidden Markov Model; GSHR, Glutathione reductase; MERA, mercuric reductase; TYTR, trypanothione reductase; DLDH, lipoamide dehydrogenase; TRXR, higher eukaryotes thioredoxin reductases; NAPE, NADH peroxidase; NAOX, NADH oxidase; NDH-2, NADH dehydrogenase-2; TRXB, prokaryotes, archaea and lower eukaryotes thioredoxin reductases; AHPF, bacterial alkyl hydroperoxide reductases; DHNA, NADH dehydrogenases or alkyl hydroperoxide reductases.

## Authors' contributions

CLA carried out data acquisition and analysis, drafted and revised the manuscript; VAR participated in the interpretation and analysis of data and revised the manuscript; RNF and JDLR participated in the design of the study, analysis of data, wrote and revised the manuscript critically; RCH conceived the study, performed the general supervision of the work and carried out the data analysis. All authors approved the final manuscript.

## Supplementary Material

Additional file 1 BLOCKS' representations of the PNDR class I fingerprint Click here for file

Additional file 2 BLOCKS' representations of the PNDR class II fingerprint Click here for file

Additional file 3 BLOCKS' representations of the PNDR class III fingerprint Click here for file

Additional file 4 BLOCKS' representations of the PNDR class IV fingerprint Click here for file
